# Comparative Assessment of Large Language Models in Optics and Refractive Surgery: Performance on Multiple-Choice Questions

**DOI:** 10.3390/vision9040085

**Published:** 2025-10-09

**Authors:** Leah Attal, Elad Shvartz, Alon Gorenshtein, Shirley Pincovich, Daniel Bahir

**Affiliations:** 1 Azrieli Faculty of Medicine, Bar Ilan University, Ramat-Gan 5290002, Israel; eladshvartz@gmail.com (E.S.); a_gorenshtein@rambam.health.gov.il (A.G.); shirleyp50@gmail.com (S.P.); bahirdaniel@gmail.com (D.B.); 2 Laniado Hospital, Netanya 4244916, Israel; 3 Kaplan Medical Center, Rehovot 7661041, Israel; 4 Department of Neurology, Rambam Health Care Campus, Haifa 3109601, Israel; 5 AI in Neurology Laboratory, Ruth and Bruce Rapaport Faculty of Medicine, Technion Institute of Technology, Haifa 3525408, Israel; 6 Ophthalmology Department, Galilee Medical Center, Nahariya 2222605, Israel; 7 Ophthalmology Department, Tzafon Medical Center, Poriya 15208, Israel

**Keywords:** artificial intelligence, large language models, ChatGPT, optics, multiple-choice questions, medical education

## Abstract

This study aimed to evaluate the performance of seven advanced AI Large Language Models (LLMs)—ChatGPT 4o, ChatGPT O3 Mini, ChatGPT O1, DeepSeek V3, DeepSeek R1, Gemini 2.0 Flash, and Grok-3—in answering multiple-choice questions (MCQs) in optics and refractive surgery, to assess their role in medical education for residents. The AI models were tested using 134 publicly available MCQs from national ophthalmology certification exams, categorized by the need to perform calculations, the relevant subspecialty, and the use of images. Accuracy was analyzed and compared statistically. ChatGPT O1 achieved the highest overall accuracy (83.5%), excelling in complex optical calculations (84.1%) and optics questions (82.4%). DeepSeek V3 displayed superior accuracy in refractive surgery-related questions (89.7%), followed by ChatGPT O3 Mini (88.4%). ChatGPT O3 Mini significantly outperformed others in image analysis, with 88.2% accuracy. Moreover, ChatGPT O1 demonstrated comparable accuracy rates for both calculated and non-calculated questions (84.1% vs. 83.3%). This is in stark contrast to other models, which exhibited significant discrepancies in accuracy for calculated and non-calculated questions. The findings highlight the ability of LLMs to achieve high accuracy in ophthalmology MCQs, particularly in complex optical calculations and visual items. These results suggest potential applications in exam preparation and medical training contexts, while underscoring the need for future studies designed to directly evaluate their role and impact in medical education. The findings highlight the significant potential of AI models in ophthalmology education, particularly in performing complex optical calculations and visual stem questions. Future studies should utilize larger, multilingual datasets to confirm and extend these preliminary findings.

## 1. Introduction

The use of artificial intelligence (AI) in medical education has significantly expanded in recent years, with remarkable progress in processing medical knowledge, interpreting images, and supporting clinical decision-making. Studies have shown that large language models (LLMs), such as ChatGPT and Gemini, can successfully pass medical certification exams with accuracy rates approaching those of human physicians [1,2]. In ophthalmology, AI has been applied in various areas, including automated image analysis for retinal diseases [3], as well as surgical planning for complex cases such as retinal detachment [4] and glaucoma [5]. Thanks to its ability to synthesize vast amounts of medical literature, identify patterns in patient data, and provide differential diagnoses, AI appears to be a promising tool in both clinical practice and medical education [6,7,8]. Indeed, AI has demonstrated the ability to answer multiple-choice questions (MCQs) with accuracy across a variety of medical specialties [9], from orthopedics [10] to endocrinology [11], including ophthalmology [2,12,13].

MCQs are a cornerstone of medical education and evaluation, offering a standardized and objective method to assess knowledge across various disciplines, including ophthalmology [14,15]. In the subspecialty of optics and refractive surgery, MCQs are essential tools for testing understanding of complex optical principles, advanced surgical techniques, and patient management strategies. However, in this particular field, AI faces significant challenges. Previous studies have highlighted substantial disparities in AI performance across ophthalmology subspecialties [1,12,13,16]. Notably, ChatGPT 4o and Gemini Advanced have shown lower accuracy rates when answering MCQs related to optics compared to subjects like retina [1,12,16]. One of the main limitations observed is the difficulty in accurately performing optical calculations, a fundamental aspect of both theoretical and clinical ophthalmology.

This study primarily aims to evaluate the accuracy of the latest AI chatbots on the market (ChatGPT 4o, O3 Mini, O1, DeepSeek V3, R1, Gemini 2.0 Flash, and Grok-3) in their ability to accurately answer MCQs related to optics and refractive surgery.

By examining their consistency, reasoning, and performance based on question type (text, image, calculation), this study aims to identify the strengths, limitations, and potential of these AI models to support medical education and exam preparation in ophthalmology. A central question is whether these models can demonstrate sufficient accuracy to suggest a possible role as supportive tools for exam preparation. Although this work does not directly evaluate learning outcomes, the findings may provide valuable guidance for educators and trainees by identifying which models are most reliable in specific domains. In this way, the study can inform the integration of LLMs into ophthalmology education as complementary resources, helping residents to practice questions more effectively, review explanations, and approach complex concepts with greater clarity.

## 2. Methods

### 2.1. Large Language Model-Based Software

This study evaluated the performance and accuracy of seven leading large language models (LLMs): ChatGPT 4o, ChatGPT O3 Mini, ChatGPT O1, DeepSeek V3, DeepSeek R1, Gemini 2.0 Flash, and Grok-3. These models were selected based on their advanced capabilities in natural language processing, reasoning, and image interpretation. ChatGPT 4o, which was the latest version from OpenAI at the time of manuscript preparation, was tested alongside the O3 Mini and O1 models, which feature reasoning capabilities compared to their predecessors. DeepSeek V3 and DeepSeek R1, developed in China, were included for their computational strength in handling complex medical data, while Gemini 2.0 Flash, Google’s advanced AI platform, was selected for its speed and scalability in providing rapid responses to diverse queries. Lastly, Grok-3, developed by xAi, was chosen for its integration of real-time data retrieval and context-driven reasoning, yielding insights into its applicability in a clinical setting.

### 2.2. Evaluation Dataset

A set of approximately 134 MCQs focusing specifically on optics and refractive surgery was submitted to the seven AI platforms. Among these, 117 questions consisted solely of text, 44 involved calculations, and 17 incorporated images. Each question presented four possible answers, with only one being correct. Furthermore, accuracy was defined as the proportion of accurately solved MCQs by each AI model.

The questions were sourced from Israeli board examinations conducted between 2020 and 2024, which are publicly accessible through the official website of the Israeli residency program [17]. They were meticulously crafted by experts in optics and refractive surgery and subsequently reviewed by other specialists to ensure accuracy and relevance.

In order to assess the accuracy and performance of these AI platforms in their native language, the models have been trained upon (English), all the questions were translated from Hebrew into English. This translation was conducted by a bilingual expert proficient in both languages. Therefore, the translation quality was entirely dependent on the translator’s expertise. No back-translation process was used.

Each question was input into the various AI platforms for evaluation as a standalone prompt, consisting solely of its text and, if applicable, associated images, without any additional instructions. Additionally, all questions were manually submitted to each chatbot interface, without the use of automated scripts or application programming interfaces (APIs). For questions that included associated images, the original figures from the Israeli national ophthalmology examination were directly copied and pasted into the input field of the LLMs, alongside the corresponding text of the question. This ensured that both the visual and textual components of the item were presented to the models exactly as they appeared in the source exam. To replicate as closely as possible the typical behavior of a resident during exam preparation, all questions were deliberately entered manually without any additional instructions. In cases where a model did not provide an answer on its first attempt, the question was resubmitted. When the AI suggested multiple possible answers, it was prompted to select only one correct response. In instances where the platform refrained from answering due to ethical concerns, the chatbot was informed that the responses were solely for educational and informational purposes.

Then, the responses generated by the chatbots were assessed using the correction grids available on the official Israeli residency program website. While the Basic and Clinical Science Course (BCSC) was used to evaluate the relevance of the questions and the accuracy of the answers generated by the chatbots, the only content directly input into any of the chatbots came from the Israeli board certification exams. BCSC content was not placed into any chatbot.

### 2.3. Statistical Analysis

The dataset was derived from six separate Israeli national ophthalmology board examinations administered between 2020 and 2024. In this study, we define an “exam” as the set of questions originating from one of these individual examinations. For the Kruskal–Wallis analysis, each model’s performance on each exam was summarized as an exam-level score (the proportion of correctly answered questions within that exam). This approach yielded six independent data points per model, enabling comparison of accuracy distributions across models while accounting for exam-to-exam variability.

Overall performance across all AI models was evaluated using the Kruskal–Wallis test. When significant differences were identified, Dunn’s post hoc tests were conducted, with Holm correction applied to control the familywise error rate while preserving reasonable statistical power. To complement the rank-based analysis, we also compared models using Chi-square tests of independence on their overall accuracy proportions. Analyses were performed on pooled question-level data, comparing total counts of correct versus incorrect responses between models. To explore performance within specific subsets of data, such as comparing Optics versus Refractive Surgery, or ChatGPT O1 against other models within individual topics, we used Chi-square tests. These were appropriate for data structured as categorical outcomes (correct vs. incorrect), allowing us to test whether the proportion of correct answers differed significantly between groups. For 2 × 2 contingency tables, we also calculated Phi (Φ) as a measure of effect size to quantify the strength of association between model identity and outcome. This approach was chosen due to the limited number of questions per topic, which would have reduced the reliability of rank-based methods like Kruskal–Wallis. This provides a more appropriate and stable method for evaluating performance differences in these smaller subsets. The Kruskal–Wallis approach leveraged the full distribution of exam scores, offering a more detailed view of each model’s consistency and relative ranking. Post hoc analysis further allowed us to identify which specific models outperformed others, thus supporting conclusions about overall performance.

A two-tailed significance level of α = 0.05 was used throughout the analyses. *p*-values below this threshold were considered statistically significant. For post hoc pairwise comparisons, *p*-values were adjusted using the Holm correction, and adjusted values were interpreted against the same α = 0.05 threshold.

All statistical analyses were performed using JASP version 0.19.2 (JASP Team, Amsterdam, The Netherlands), which provides a graphical interface for R-based statistical routines, together with the Analysis ToolPak add-in for Microsoft Excel (Microsoft 365 version).

## 3. Results

All results are presented in Table A1.

Questions that remained unanswered by the AI were excluded from the statistical analysis. Response rates varied across models: ChatGPT models and Grok-3 answered 100% of the questions, Gemini 2.0 Flash achieved 98.5%, and the DeepSeek models answered 87.3%, with most omissions attributable to their inability to process image-based questions.

### 3.1. Overall Performance

**Figure 1:**
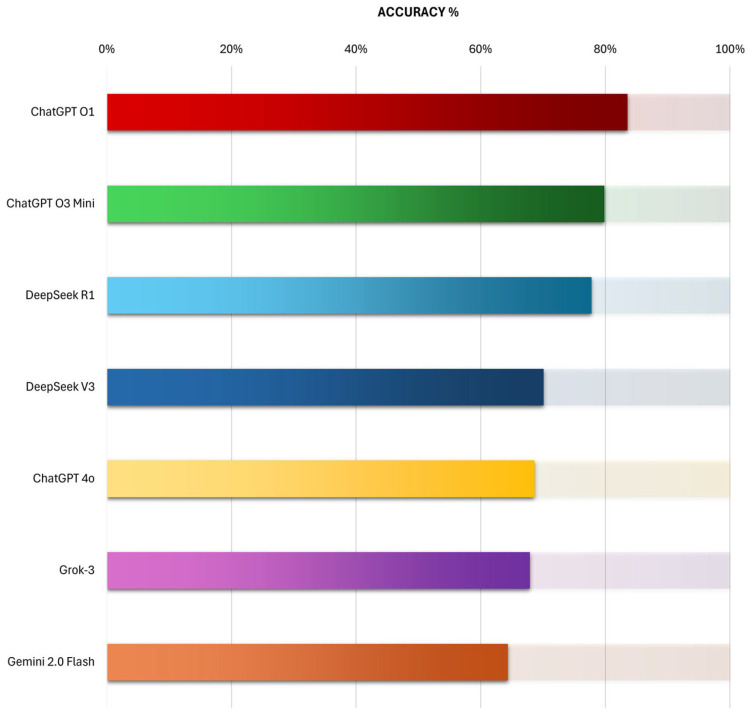
Accuracy Comparison of AI Chatbots on Optics and Refractive Surgery Multiple-Choice Questions (MCQs).

The Kruskal–Wallis test revealed a statistically significant difference in performance (*p* = 0.007), with a moderate to large effect size (η^2^ = 0.332, 95% CI: 0.17–0.67). ChatGPT O1 demonstrated the highest average rank across exams and achieved the highest overall accuracy (84%), compared to ChatGPT O3 Mini (80%), DeepSeek R1 (78%), DeepSeek V3 (70%), ChatGPT 4o (69%), Grok-3 (68%), and Gemini 2.0 Flash (64%) (Figure 1, Table A1).

As a descriptive reference, overall accuracy percentages are reported here, though they were not used directly in the Kruskal–Wallis test, which was based on exam-level scores. The Chi-square comparison, however, used these percentages as proportions of overall correct versus incorrect answers for each model.

In pairwise post hoc testing using Dunn’s test, ChatGPT O1 significantly outperformed DeepSeek V3 (*p* = 0.037), ChatGPT 4o (*p* = 0.014), Gemini 2.0 Flash (*p* = 0.001), and Grok-3 (*p* = 0.008). This was further demonstrated by the complementary Chi-square comparisons (Table A1) highlighting significant performance differences in these models with *p* values 0.011, 0.005, 0.001, and 0.003, respectively.

However, correction using the Bonferroni and Holm methods increased the threshold for significance, resulting in only a single significant comparison—ChatGPT O1 against Gemini 2.0 Flash, with corrected p_holm_ = 0.024. No other comparisons reached statistical significance after correction, although the comparisons between ChatGPT O1 and Grok-3 (p_holm_ = 0.16, r = 0.83) and ChatGPT O1 and ChatGPT 4o (p_holm_ = 0.25, r = 0.78) demonstrated large effect sizes despite not maintaining significance after adjustment. In contrast, the comparison between ChatGPT O1 and ChatGPT O3 Mini showed only a small effect size (p_holm_ = 1.0, r = 0.25), indicating that their performance was relatively similar. These findings suggest that while only one pairwise difference was statistically robust, ChatGPT O1 consistently achieved the highest average rank and demonstrated practical superiority over several models.

### 3.2. Performance by Need for Calculations

**Figure 2:**
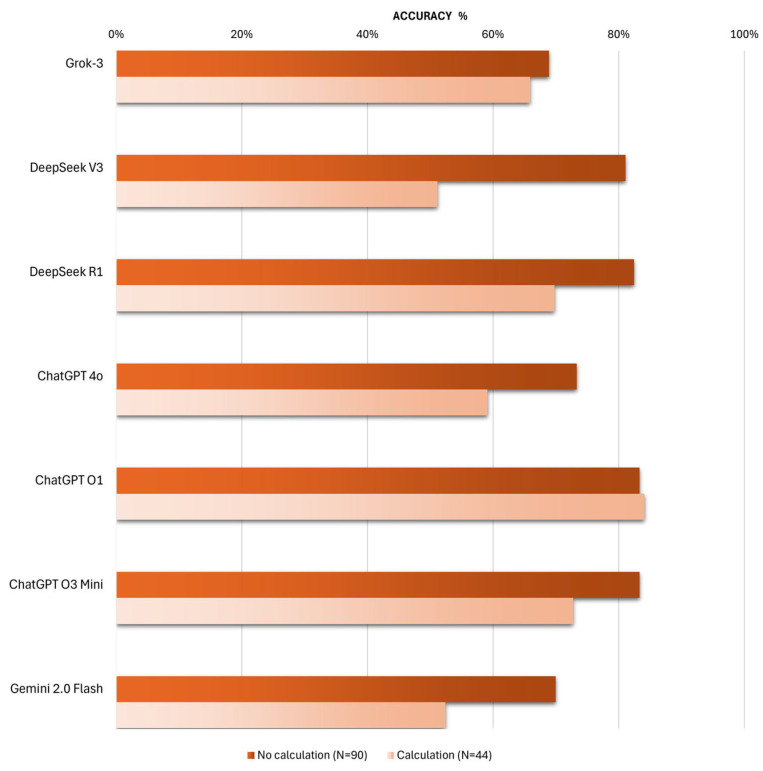
Comparison of AI Chatbot Accuracy on Optics and Refractive Surgery MCQs With and Without Calculation Requirements.

One of the most intriguing aspects of this research lies in the analysis of the calculation abilities of the various chatbots, and this aspect is exposed in Figure 2. To further illustrate this point, a representative calculation-based question, together with the corresponding LLM responses, has been included in the Supplementary Material.

Unsurprisingly, most chatbots provide more accurate responses to questions that do not involve calculations, with the exception of ChatGPT O1.

Indeed, ChatGPT O1 stands out with impressive calculation skills, achieving an accuracy of 84% for calculation-based questions, compared to an accuracy of 83% for questions that do not require calculations.

In comparison, the other models exhibit significantly lower accuracy in handling calculations, with ChatGPT O3 Mini ranking second at 73% accuracy (*p*-value 0.195) and DeepSeek R1 ranking third with 70% accuracy (*p*-value 0.11).

Statistical analysis confirms that ChatGPT O1 demonstrates superior performance in addressing calculation-based questions compared to Grok-3, which achieves 66% accuracy (*p*-value 0.049), ChatGPT 4o with 59% (*p*-value 0.009), and Gemini 2.0 Flash with 52% accuracy (*p*-value 0.002).

Surprisingly, DeepSeek V3 ranks last with an accuracy of 51% (*p*-value 0.001), highlighting a notable difference compared to the R1 version, which exhibits more advanced reasoning capabilities.

### 3.3. Performance by Subspecialty

**Figure 3:**
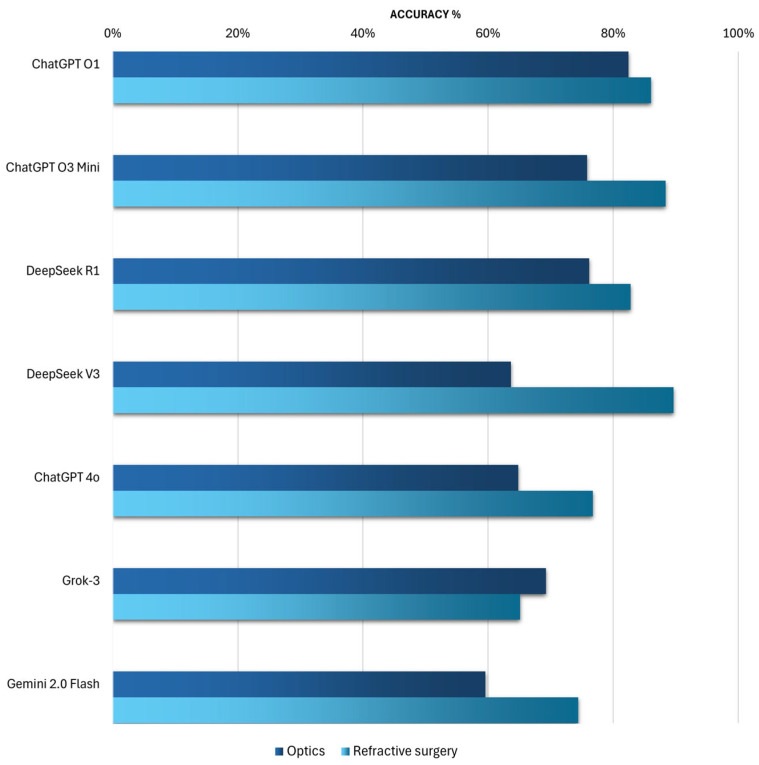
Comparison of AI Chatbot Accuracy Between Optics and Refractive Surgery Multiple-Choice Questions.

Statistical analysis revealed the following findings (Figure 3).

Nearly all chatbots demonstrated higher accuracy for questions related to refractive surgery compared to those concerning optics, with the exception of Grok-3.

For questions related to optics, ChatGPT O1 stands out with an accuracy of 82%. Following this, DeepSeek R1 achieves 76% accuracy (*p*-value 0.30), as ChatGPT O3 Mini (*p*-value 0.27). Comparisons with DeepSeek R1 and ChatGPT O3 Mini did not reach statistical significance, indicating that their performance was not substantially different from ChatGPT O1. The small effect sizes in these cases (Φ = 0.078 for DeepSeek R1 and Φ = 0.081 for ChatGPT O3 Mini) suggest that any observed differences were likely due to random variation rather than true performance gaps. Statistically, ChatGPT O1 significantly outperforms Grok-3 (69%), the previous model ChatGPT 4o (65%), DeepSeek V3 (64%), and Gemini 2.0 Flash (60%).

For questions related to refractive surgery, ChatGPT O1 is surpassed by DeepSeek V3, which achieves an exceptional accuracy of 90% (*p*-value 0.65), and by ChatGPT O3 Mini, which reaches 88% accuracy (*p*-value 0.74). ChatGPT O1 ranks third in this category with an accuracy of 86%, followed by DeepSeek R1, which, this time, demonstrates lower accuracy than its counterpart DeepSeek V3, with an accuracy of 83%.

At the lower end of the ranking, ChatGPT 4o achieves 77% accuracy (*p*-value 0.27), Gemini 2.0 Flash ranks second to last with 74% accuracy (*p*-value 0.18), and Grok-3 ranks last with 65% correct answers (*p*-value 0.02).

While a statistical difference was observed between ChatGPT O1 and Grok-3, all other comparisons involving ChatGPT O1 showed only small effect sizes, suggesting that their performance was not meaningfully different from that of ChatGPT O1.

### 3.4. Performance by Question Format

**Figure 4:**
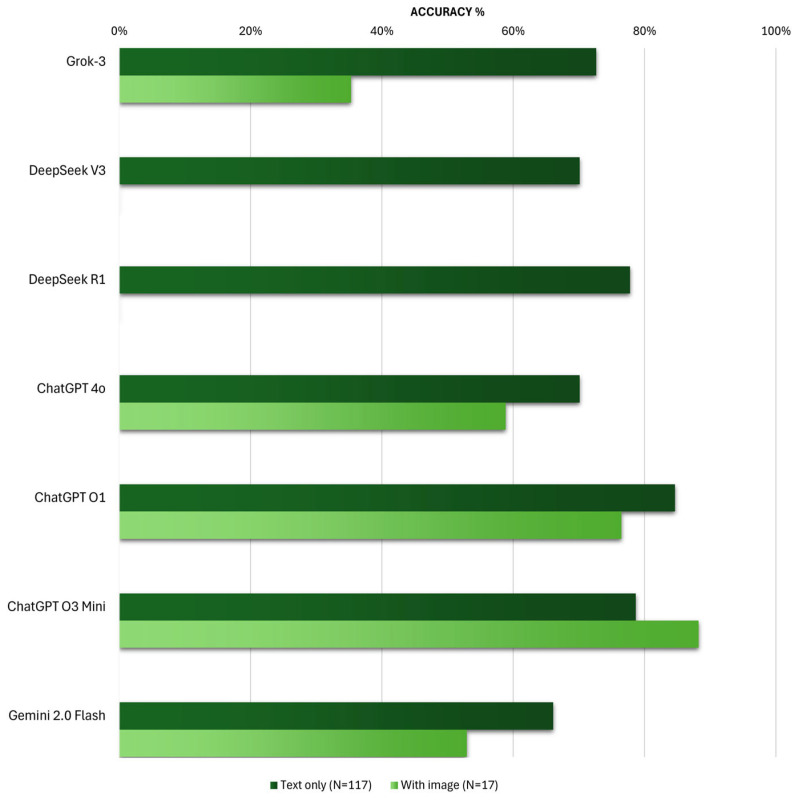
Comparison of AI Chatbot Accuracy on Multimodal Benchmarks with and Without Image Input.

Regarding the ability of chatbots to handle questions involving image analysis, all models demonstrate reduced performance, except for ChatGPT O3 Mini (Figure 4).

For text-only questions, ChatGPT O1 once again claims the top position with an accuracy of 85%, followed by ChatGPT O3 Mini with 79% accuracy (*p*-value 0.24) and DeepSeek R1 achieving 78% (*p*-value 0.18). In comparison, and with statistical significance, the other models prove to be less effective than ChatGPT O1, with Grok-3 achieving approximately 73% (*p*-value 0.03), while DeepSeek V3 and ChatGPT 4o display identical accuracy rates of 70% (*p*-value 0.008). Gemini Flash 2.0 ranks lowest with an accuracy of 66% (*p*-value 0.001).

For questions involving image analysis, it is important to emphasize that the DeepSeek models were excluded from statistical analysis due to their inability to process this format. This time, ChatGPT O3 Mini outperforms ChatGPT O1 with an accuracy of 88% (*p*-value 0.37). Following this, ChatGPT O1, with an accuracy of 77%, surpasses the other models. ChatGPT 4o ranks third with 59% accuracy (*p*-value 0.27), followed by Gemini 2.0 Flash (53% accuracy, *p*-value 0.15). It is also important to note that, although the difference in performance between Gemini 2.0 Flash and ChatGPT O1 did not reach statistical significance, the moderate effect size observed indicates a potentially meaningful difference in model accuracy that should be interpreted with caution considering the sample size. Furthermore, at the bottom of the ranking, Grok-3 struggles to achieve 35% correct answers (*p*-value 0.016), highlighting its markedly superior performance in handling text-based questions compared to those requiring image analysis.

## 4. Discussion

### 4.1. Comparative Accuracy of LLMs in Optics and Refractive Surgery

In this study, the advanced reasoning model developed by OpenAI, ChatGPT O1, achieved the highest performance, outperforming ChatGPT O3 Mini by nearly 4%, with the latter occupying second place. OpenAI models continue to improve over time, and the performance of their flagship model, ChatGPT O1, continues to draw attention [18]. The O1 model introduces significant advancements over prior versions of ChatGPT, particularly in its reasoning capabilities. This enhancement allows the model to engage in cognitive processes prior to task execution [19]. These allow ChatGPT O1 to tackle more complex tasks, such as managing intricate multi-systemic diseases, discovering genetic disorders, and supporting medical research [20].

Prior studies have shown that ChatGPT O1 achieves high accuracy rates in complex fields such as psychiatric cases, understanding of ethical issues, and the Japanese national examination for physical therapy [21,22]. The studies have shown high discrepancy between ChatGPT 4o and O1, a 41% gap [22].

ChatGPT O3 Mini also demonstrates strong performance, securing second place, while ChatGPT 4o falls to fourth position. Although ChatGPT 4o previously led the field of chatbots before the development of new GPT models and the launch of DeepSeek [1,12], it is now outperformed by these newer models. This decline can be attributed to ChatGPT 4o’s lack of advanced reasoning abilities, rendering it less suitable for complex critical tasks.

A genuine breakthrough in artificial intelligence, DeepSeek, particularly DeepSeek R1, lives up to its promises with performance slightly lower than that of ChatGPT O3 Mini. Despite being a relatively new Chinese chatbot released only on 25 January 2025, and developed with a budget significantly smaller than that of ChatGPT, its results are impressive [23].

According to Zhou and Pan, DeepSeek R1 also produces clearer explanations when generating educational material for spinal cord surgeries compared to GPT O3 Mini, which could help improve patient adherence, reduce anxiety, and ultimately achieve better postoperative outcomes [24].

Grok-3, still scarcely studied in recent research, performs less effectively than its competitors and still has room for improvement. As for Gemini 2.0 Flash, its ranking at the bottom of the list is unsurprising, corroborating findings from our previous studies [1,12].

It should be noted that ChatGPT models and Grok-3 provided responses to 100% of the questions, Gemini 2.0 Flash answered 98.5%, and DeepSeek models answered 87.3% of the questions, primarily due to their inability to support image-based questions.

For context, when considered in relation to the standards required for board certification, the performance of the LLMs appears particularly noteworthy. The Israeli national ophthalmology exam requires a minimum passing score of 65% across all subspecialties, including retina, cornea, glaucoma, pediatric ophthalmology, optics, refractive surgery, etc. Applying this benchmark solely to the domains analyzed in the present study, all models except Gemini 2.0 Flash would have achieved a passing score, with Gemini missing the cut-off by only 0.6%. These results illustrate how closely the models’ accuracy approaches certification expectations, while at the same time emphasizing that such performance within a limited subset of topics cannot be equated with success on the full board examination.

### 4.2. Evaluation of LLMs for Calculation-Based Questions

In this study, the major aim was to assess whether chatbots can perform complex calculations, thereby addressing ophthalmology-related optics questions. This inquiry is particularly relevant, as demonstrated by the present study, where nearly all chatbots, except ChatGPT O1, exhibit higher accuracy when responding to questions that do not involve calculations compared to those that do.

Remarkably, artificial intelligence has shown substantial progress in this area of calculation processing, with ChatGPT O1 achieving nearly the same accuracy for questions involving calculations as it does for non-calculation-based questions. Notably, as ChatGPT models continue to advance, their calculation abilities have markedly improved. For instance, the performance gap between questions with and without calculations, which was approximately 14% for the previous model ChatGPT 4o, has narrowed to just under 11% for ChatGPT O3 Mini and has been entirely eliminated by ChatGPT O1.

Similarly, DeepSeek R1 significantly reduces its performance gap for calculation-based versus non-calculation-based questions compared to its less sophisticated predecessor, DeepSeek V3. Interestingly, models designed to provide explanations of their reasoning processes, such as ChatGPT O1, ChatGPT O3 Mini, and DeepSeek R1, tend to exhibit the smallest performance gaps between these two categories of questions.

Although Grok-3 does not excel in overall accuracy, it is noteworthy that this model processes questions with and without calculations in a relatively consistent manner, with only a three-percentage-point difference in accuracy between the two categories.

These findings suggest that artificial intelligence is rapidly improving in handling optics-related calculations, with performance levels approaching those achieved for non-calculation questions. This observation is particularly encouraging for ophthalmology residents learning optics. They can now be assured that if they seek a cost-effective tool for studying optics, ChatGPT O1 is currently the most adequate chatbot to meet their needs.

### 4.3. Comparative Performance of LLMs Across Subspecialties

For the subspecialties analyzed in this study, a significant improvement in the accuracy of newly developed chatbots compared to earlier AI models is evident.

Regarding questions related to optics, ChatGPT O1 achieves an accuracy of 82%. Even DeepSeek V3 and R1 surpass older chatbot models in precision, as demonstrated in this study and supported by numerous other investigations on the topic [25,26]. Previous studies indicated that earlier versions of the chatbot achieved accuracy rates ranging from 38% to 69% [1,12,27].

For refractive surgery-related questions, this field demonstrated higher accuracy rates. In our study, DeepSeek V3 achieved an impressive 90%, ChatGPT O3 Mini 88%, and ChatGPT O1 86%. These results signify a significant advancement compared to earlier studies, which reported accuracy rates ranging from 48% to 77% [1,12,27]. Such findings may suggest that in the subspecialties of optics and refractive surgery, notable advancements have been achieved by existing AI systems.

### 4.4. Evaluation of Image Processing Capabilities

Regarding image processing, the latest chatbot models demonstrate significantly enhanced capabilities compared to their predecessors. In the present study, ChatGPT O3 Mini achieved the highest accuracy of 88% in image analysis, while ChatGPT O1 reached 77%. The reason behind it is due to the fact that ChatGPT O3 Mini improved its visual interpretation capabilities. ChatGPT 4o demonstrated a precision of approximately 59%, a finding that aligns with our previous research [1]. These results indicate a substantial improvement over earlier chatbot models, where a combined set of four chatbots (ChatGPT 4, ChatGPT 3, Gemini, and Gemini Advanced) achieved only 42% accuracy in image-based questions [12].

Gemini has also shown progress in image interpretation, with its accuracy increasing from 34% in our prior French-language study [1] to 53% in the current evaluation. This suggests that chatbot models have undergone more extensive training in image analysis, further advancing their capabilities in this area.

DeepSeek remains the sole chatbot limited in this domain, with its inability to process images representing a notable disadvantage compared to its peers. Image interpretation is a fundamental skill in ophthalmology training, playing a crucial role in guiding clinical decision-making. A 2024 study by Hirosawa and Harada highlighted the deficiencies of earlier chatbots, such as ChatGPT 4, in image analysis and emphasized the need to enhance AI capabilities in this domain to support clinical practice [28]. The improvements observed in this study suggest that chatbot models are progressively overcoming these limitations, which may contribute to better AI-assisted clinical decision-making in the future.

### 4.5. AI and Optics: A New Era in Medical Education

The integration of AI within the field of optics is advancing at an exponential rate. AI is enhancing surgeons’ skills and providing ophthalmologists with foundational models designed for diagnosing and predicting a range of ocular diseases, utilizing multiple imaging modalities [29,30]. These tools are increasingly being incorporated into clinical practice, assisting physicians in diagnostics and alleviating their documentation burden [31,32,33]. To facilitate this clinical integration, the adoption of LLMs should commence early in medical education [34], a development already underway with the introduction of AI-focused courses in medical schools [34]. Our research highlights the critical role these studies play in demonstrating the evolving reliability of LLMs as educational resources for medical students and practitioners. Historically, medical professionals have relied on textbooks and internet searches to address clinical inquiries. LLMs now markedly reduce search time, delivering high-quality information with greater efficiency [35]. The primary objective of our study was to evaluate whether LLMs can accurately respond to calculation-based questions, a known challenge within LLM systems, which have traditionally excelled at text prediction but faltered at numerical computations. Our findings indicate that LLMs like ChatGPT O1 demonstrate comparable accuracy in solving calculation problems to that of non-calculation questions. This suggests that medical students and ophthalmologists can effectively incorporate these AI models into their educational and clinical routines, aiding them in addressing complex mathematical queries encountered in their practice. As accessible, low-cost, and continuously available systems, they could provide residents with personalized educational assistance, helping them understand complex concepts more effectively [36]. Furthermore, chatbots could offer a safe and welcoming learning environment where residents feel free to ask questions they might hesitate to present to their supervisors. This approach would allow senior physicians to focus on addressing the most challenging inquiries, thus optimizing their valuable time.

Future improvements to these models could include categorizing questions by difficulty level, citing reliable references, and accurately interpreting medical images. Such advancements could transform medical education by offering innovative strategies to enhance learning experiences and better prepare future physicians.

However, incorporating AI chatbots in education comes with significant limitations. A primary concern is their tendency for “hallucinations,” where they produce plausible but incorrect information [37,38,39]. Unlike human educators, chatbots cannot recognize their uncertainty and may mislead students, creating a false sense of certainty or self-doubt. This misinformation can greatly impact clinical decision-making in medical education. While many models generate step-by-step rationales, recent evidence suggests that such explanations do not always reflect the actual computational pathways used to reach the final answer, but rather are post hoc narratives constructed to appear plausible [40,41,42]. This lack of transparency poses a particular challenge in educational contexts, where understanding the reasoning process is as critical as obtaining the correct answer. Without reliable explanations, learners may adopt inaccurate or misleading thought patterns, potentially reinforcing misconceptions. In the present study, the accuracy of responses was the primary focus, and the quality or faithfulness of model explanations was not evaluated. Future research should specifically investigate the explanatory reliability of LLMs, with the aim of determining whether their outputs can genuinely support the development of critical thinking skills and deeper conceptual understanding in ophthalmology education.

Additionally, while AI models continuously integrate new data, they may retain outdated or non-professional information, as they struggle to differentiate between academic and non-academic sources [43] unless specifically trained. Lastly, AI lacks human empathy, which is vital in medical education, potentially affecting the overall quality of learning experiences [30,44].

### 4.6. Limitations

All the multiple-choice questions included in this study were originally composed in Hebrew and subsequently translated into English by a bilingual expert. While great care was taken to preserve the integrity of the content, this translation process could introduce potential biases or subtle inaccuracies. Another important limitation of this study is the relatively small number of questions included in the dataset, particularly for subsets involving image analysis. Future studies with larger datasets are required to confirm these preliminary findings and to perform more robust statistical comparisons.

## 5. Conclusions

This study demonstrates that LLMs achieve high accuracy when answering MCQs in optics and refractive surgery, with ChatGPT O1 performing best overall, especially in complex optical calculations, ChatGPT O3 Mini excelling in image interpretation, and DeepSeek V3 showing strong precision in refractive surgery. These findings suggest the potential utility of such models in medical education and exam preparation. However, strong performance on MCQs alone does not establish direct educational value, and further studies are needed to evaluate their impact on learning outcomes, usability in training contexts, and integration into ophthalmology curricula.

## Data Availability

All data analyzed in this study are included in this article. Further inquiries can be directed to the corresponding author.

## Supplementary Materials

The following supporting information can be downloaded at: https://www.mdpi.com/article/10.3390/vision9040085/s1, File S1: Calculation Question Example and LLM Responses.

## Appendix A

**Table A1:** Comparison of AI Chatbot Accuracy on Optics and Refractive Surgery Questions by Model, Topic, Input Modality, and Calculation Requirement.

Baseline	Accuracy	Contender	Accuracy	All Responses	Odds Ratio	95% CI	*p*-Value	Phi Φ
Overall comparison								
ChatGPT O1	112/134 (83.6%)	Grok-3	91/134 (67.9%)	268	0.42	0.23–0.75	0.003	0.19
		DeepSeek V3	82/117 (70.1%)	251	0.47	0.25–0.84	0.011	0.17
		DeepSeek R1	91/117 (77.8%)	251	0.69	0.37–1.29	0.244	0.08
		ChatGPT 4o	92/134 (68.7%)	268	0.44	0.24–0.77	0.005	0.18
		ChatGPT O3 Mini	107/134 (79.9%)	268	0.78	0.42–1.45	0.430	0.05
		Gemini 2.0 Flash	85/132 (64.4%)	266	0.36	0.2–0.63	<0.001	0.22
Comparison by topics								
ChatGPT O1 Optics	75/91 (82.4%)	Grok-3	63/91 (69.2%)	182	0.48	0.24–0.97	0.038	0.16
		DeepSeek V3	56/88 (63.6%)	179	0.38	0.19–0.75	0.005	0.22
		DeepSeek R1	67/88 (76.1%)	179	0.69	0.33–1.41	0.300	0.08
		ChatGPT 4o	59/91 (64.8%)	182	0.40	0.2–0.78	0.008	0.20
		ChatGPT O3 Mini	69/91 (75.8%)	182	0.67	0.32–1.38	0.274	0.09
		Gemini 2.0 Flash	53/89 (59.6%)	180	0.32	0.16–0.62	<0.001	0.26
ChatGPT O1 Refractive	37/43 (86%)	Grok-3	28/43 (65.1%)	86	0.31	0.1–0.88	0.024	0.25
		DeepSeek V3	26/29 (89.7%)	72	1.41	0.32–6.14	0.650	0.06
		DeepSeek R1	24/29 (82.8%)	72	0.78	0.21–2.84	0.704	0.05
		ChatGPT 4o	33/43 (76.7%)	86	0.54	0.18–1.63	0.268	0.12
		ChatGPT O3 Mini	38/43 (88.4%)	86	1.24	0.35–4.39	0.747	0.04
		Gemini 2.0 Flash	32/43 (74.4%)	86	0.48	0.16–1.42	0.176	0.15
Comparison by calculation-based questions								
ChatGPT O1 No calculation	75/90 (83.3%)	Grok-3	62/90 (68.9%)	180	0.45	0.22–0.9	0.024	0.17
		DeepSeek V3	60/74 (81.1%)	164	0.86	0.38–1.91	0.707	0.03
		DeepSeek R1	61/74 (82.4%)	164	0.94	0.41–2.12	0.879	0.02
		ChatGPT 4o	66/90 (73.3%)	180	0.55	0.27–1.14	0.104	0.13
		ChatGPT O3 Mini	75/90 (83.3%)	180	1.00	0.46–2.19	1.000	0.00
		Gemini 2.0 Flash	63/90 (70%)	180	0.47	0.23–0.95	0.035	0.16
ChatGPT O1 Calculation		Grok-3	29/44 (65.9%)	88	0.37	0.13–1.01	0.049	0.21
		DeepSeek V3	22/43 (51.2%)	87	0.20	0.07–0.54	0.002	0.36
		DeepSeek R1	30/43 (69.8%)	87	0.44	0.15–1.23	0.113	0.18
		ChatGPT 4o	26/44 (59.1%)	88	0.28	0.1–0.75	0.010	0.28
		ChatGPT O3 Mini	32/44 (72.7%)	88	0.51	0.18–1.44	0.196	0.14
		Gemini 2.0 Flash	22/42 (52.4%)	86	0.21	0.08–0.57	0.002	0.35
Comparison of text-only vs. image-based questions								
ChatGPT O1 Text only	99/117 (84.6%)	Grok-3	85/117 (72.6%)	234	0.49	0.25–0.92	0.026	0.15
		DeepSeek V3	82/117 (70.1%)	234	0.43	0.22–0.81	0.008	0.18
		DeepSeek R1	91/117 (77.8%)	234	0.64	0.33–1.24	0.181	0.09
		ChatGPT 4o	82/117 (70.1%)	234	0.43	0.22–0.81	0.008	0.18
		ChatGPT O3 Mini	92/117 (78.6%)	234	0.67	0.34–1.31	0.238	0.08
		Gemini 2.0 Flash	76/115 (66.1%)	232	0.36	0.19–0.67	0.002	0.22
ChatGPT O1 With Image	13/17 (76.5%)	Grok-3	6/17 (35.3%)	34	0.17	0.04–0.75	0.016	0.42
		DeepSeek V3	-	17	-	-	-	-
		DeepSeek R1	-	17	-	-	-	-
		ChatGPT 4o	10/17 (58.8%)	34	0.44	0.1–1.93	0.272	0.19
		ChatGPT O3 Mini	15/17 (88.2%)	34	2.31	0.36–14.72	0.369	0.16
		Gemini 2.0 Flash	9/17 (52.9%)	34	0.35	0.08–1.51	0.152	0.25
