# Supplementary material for: Comparative Assessment of Large Language Models in Optics and Refractive Surgery: Performance on Multiple-Choice Questions
Source: Vision (Basel). 2025 Oct 9;9(4):85. doi: 10.3390/vision9040085 (PMC12550897; doi:10.3390/vision9040085)
Supplement: Supplementary file 1 [file vision-09-00085-s001.zip › Supplementary Material Calculation Question Example.pdf]

Supplementary Material - Calculation Question Example and LLM Responses

Chatgpt O3-MINI : Correct answer

17. What is the size of the smallest letter that a person with visual acuity LogMar0.3 will recognize from 3 meters?
A- 4.45 mm
B- 8.9 mm
C- 18.0 mm
D- 0.34 cm

Raisonné sur la reconnaissance de lettres par acuité visuelle pendant 15 secondes

To determine the smallest letter a person with a LogMAR of 0.3 can recognize from 3 meters, we follow these steps:

1. **Determine the Minimum Angle of Resolution (MAR):**
   The LogMAR scale is defined as:

$$\text{MAR (in minutes of arc)} = 10^{\text{logMAR}}$$

   For a LogMAR of 0.3:

$$\text{MAR} = 10^{0.3} \approx 2 \text{ minutes of arc}$$

---

2. **Calculate the Letter's Overall Angular Size:**
   Standard optotypes (letters on a vision chart) are designed so that the entire letter subtends 5 times the MAR. Therefore, the angular size of the letter is:

$$5 \times 2 = 10 \text{ minutes of arc}$$

3. **Convert the Angular Size to Radians:**

$$10 \text{ minutes} = \frac{10}{60} \text{ degrees} \approx 0.16667^\circ$$

   Converting degrees to radians:

$$0.16667^\circ \times \frac{\pi}{180} \approx 0.0029089 \text{ radians}$$

4. **Compute the Letter's Size at 3 Meters:**
   Using the small-angle approximation:

$$\text{Letter height} = \text{distance} \times \text{angle in radians}$$

   For a distance of 3 meters (3000 mm):

$$\text{Letter height} \approx 3000\,\text{mm} \times 0.0029089 \approx 8.73\,\text{mm}$$

   This value rounds to approximately 8.9 mm.

Given the answer choices, the correct answer is:

B- 8.9 mm

ChatGPT O1 : Correct answer

17. What is the size of the smallest letter that a person with visual acuity LogMar0.3 will recognize from 3 meters?
A- 4.45 mm
B- 8.9 mm
C- 18.0 mm
D- 0.34 cm

Raisonnement terminé pendant 30 secondes

First, recall that a visual acuity of LogMAR 0.0 corresponds to the classic “6/6” (or 20/20) vision, and LogMAR 0.3 corresponds roughly to “6/12” vision. The key point is that a LogMAR 0.3 acuity means the person’s minimum angle of resolution (MAR) is 2 minutes of arc instead of 1 minute (which is normal).

Step-by-step reasoning

1. **Minimum Angle of Resolution (MAR)**
   - LogMAR 0.0 ⇒ MAR = 1 minute of arc
   - LogMAR 0.3 ⇒ MAR = 2 minutes of arc
2. **Height of an optotype**
   On a standard Snellen chart, the height of the letter is about 5× the MAR (the letter’s critical detail is 1× MAR, and the overall letter height subtends 5× that angle).
   - For LogMAR 0.3 (MAR = 2′), total angular subtense of the letter = 5 × 2′ = 10 minutes of arc.
3. **Converting angular size to linear size**
   The linear height H of a letter subtending θ radians at distance D is:

$$H = D \times \tan(\theta).$$

   For small angles, $\tan(\theta) \approx \theta$ (in radians). We have:
   - 10 minutes of arc = 10/60 degrees = 0.1667 degrees.
   - Convert to radians:

$$0.1667^\circ \times \frac{\pi}{180^\circ} \approx 0.00291 \text{ radians.}$$

   - Distance $D = 3$ meters = 3000 mm.

Therefore,

$$H \approx 3000\,\mathrm{mm} \times 0.00291 \approx 8.73\,\mathrm{mm}.$$

Hence, at 3 m, the smallest letter that someone with LogMAR 0.3 (6/12) vision can reliably resolve is approximately **8.7–8.9 mm** in height.

Matching to the given choices

Among the provided options:

- **A. 4.45 mm**
- **B. 8.9 mm**
- **C. 18.0 mm**
- **D. 0.34 cm (= 3.4 mm)**

The closest correct value is **8.9 mm**, which matches our calculation.

Answer: 8.9 mm (Option B)

ChatGPT 4O : Uncorrect answer

17. What is the size of the smallest letter that a person with visual acuity LogMar0.3 will recognize from 3 meters?
A- 4.45 mm
B- 8.9 mm
C- 18.0 mm
D- 0.34 cm

To determine the smallest letter size a person with **LogMAR 0.3** visual acuity can recognize from **3 meters**, we use the following relationships:

1. **LogMAR to Snellen conversion**
   - LogMAR 0.3 corresponds to a Snellen fraction of **20/40** (or **6/12** in metric units).
2. **Letter size formula**
   The height of an optotype (letter) at a given distance is calculated using the formula:

$$\text{Letter height} = \tan(5') \times \text{test distance}$$

   - **5' (arc minutes) ≈ 0.00145 radians**
   - **Snellen 20/40 (6/12)** means the letter size for **20 feet (6 meters)** is **8.9 mm**.

3. **Scaling to 3 meters**
   - Since letter size is proportional to distance, at **3 meters**, the letter size is:

$$8.9 \times \frac{3}{6} = 4.45 \text{ mm}$$

Answer:

A - 4.45 mm

Grok 3 : Correct answer

17. What is the size of the smallest letter that a person with visual acuity LogMar0.3 will recognize from 3 meters?
A- 4.45 mm
B- 8.9 mm
C- 18.0 mm
D- 0.34 cm

To determine the size of the smallest letter that a person with a visual acuity of LogMAR 0.3 can recognize from 3 meters, we need to understand the relationship between LogMAR, the minimum angle of resolution (MAR), and the physical size of the letter on a chart.

Step 1: Understanding LogMAR and MAR

- LogMAR stands for "Logarithm of the Minimum Angle of Resolution."
- A LogMAR value of 0.0 corresponds to a MAR of 1 minute of arc (standard vision, equivalent to 6/6 or 20/20), where the letter subtends 5 minutes of arc (since the letter height is typically 5 times the stroke width, which defines the MAR).
- The MAR is the smallest angle (in minutes of arc) that the eye can resolve, and LogMAR is the base-10 logarithm of this value:

$$\text{LogMAR} = \log_{10}(\text{MAR})$$

- For LogMAR 0.3:

$$\text{MAR} = 10^{0.3} \approx 1.995 \text{ minutes of arc}$$

This means the person can resolve details separated by approximately 2 minutes of arc.

Step 2: Relating MAR to Letter Size

- In visual acuity charts (like Snellen or LogMAR charts), the MAR corresponds to the stroke width or the gap size of the optotype (e.g., the gap in a Landolt C or the stroke width of a letter). The total height of the letter is conventionally 5 times the MAR, because the letter is designed to subtend 5 minutes of arc at the distance where it is just recognizable by a person with standard vision.
- At a given distance D (in meters), the physical size of the MAR (in meters) can be calculated using the small-angle approximation:

$$\text{Size of MAR (in meters)} = D \times \tan(\text{MAR in radians})$$

- Convert MAR from minutes of arc to radians:

$$1 \text{ minute of arc} = \frac{\pi}{180 \times 60} \approx 2.9089 \times 10^{-4} \text{ radians}$$

So, for MAR = 1.995 minutes of arc:

$$\text{MAR in radians} = 1.995 \times 2.9089 \times 10^{-4} \approx 5.802 \times 10^{-4} \text{ radians}$$

- At 3 meters:

$$\text{Size of MAR} = 3 \times 5.802 \times 10^{-4} \approx 0.0017406 \text{ meters} = 1.7406 \text{ mm}$$

- The letter height is 5 times the MAR size:

$$\text{Letter height} = 5 \times 1.7406 \text{ mm} \approx 8.703 \text{ mm}$$

This is the size of the smallest letter the person can recognize at 3 meters.

Step 3: Adjusting for Testing Distance

- LogMAR 0.3 corresponds to a Snellen equivalent of 6/12 (or 20/40), meaning the person can read at 6 meters what a person with standard vision (6/6) can read at 12 meters. At 3 meters, the effective acuity adjusts because the distance is halved:

$$\text{LogMAR at 3 meters} = 0.3 + \log_{10}(6/3) = 0.3 + 0.3 = 0.6$$

However, the question asks for the smallest letter recognized at 3 meters with an acuity of LogMAR 0.3 as measured at a standard distance (typically 6 meters). Thus, we use the MAR derived from LogMAR 0.3 directly at 3 meters, as calculated above.

Step 4: Compare with Options

- Calculated letter height ≈ 8.703 mm (rounds to 8.7 mm or 8.9 mm depending on precision).
- Options:
  A. 4.45 mm
  B. 8.9 mm
  C. 18.0 mm
  D. 0.34 cm (3.4 mm)

The closest match to 8.703 mm is **B. 8.9 mm**.

Final Answer: B. 8.9 mm

Deepseek V3 : Correct answer

17. What is the size of the smallest letter that a person with visual acuity LogMar0.3 will recognize from 3 meters?
A- 4.45 mm
B- 8.9 mm
C- 18.0 mm
D- 0.34 cm

To determine the size of the smallest letter that a person with a visual acuity of LogMAR 0.3 can recognize from 3 meters, we need to understand the relationship between LogMAR and letter size.

Step 1: Understand LogMAR

- **LogMAR (Logarithm of the Minimum Angle of Resolution)** is a measure of visual acuity.
- A LogMAR of 0.0 corresponds to normal vision (20/20 Snellen acuity).
- Each increase of 0.1 in LogMAR represents a decrease in visual acuity by a factor of 10^(0.1).

Step 2: Convert LogMAR to Snellen Acuity

- A LogMAR of 0.3 corresponds to a Snellen acuity of approximately 20/40.
- This means that what a person with normal vision can see at 40 feet, a person with LogMAR 0.3 can see at 20 feet.

Step 3: Calculate Letter Size

- The standard Snellen chart uses letters that are 8.73 mm tall at 20 feet for the 20/20 line.
- For 20/40 acuity, the letters need to be twice as large (since 40/20 = 2).
- Therefore, the letters should be 8.73 mm * 2 = 17.46 mm tall at 20 feet.

Step 4: Adjust for Distance

- The question asks for the letter size at 3 meters (approximately 9.84 feet).
- Since the required letter size is inversely proportional to the distance, we can use the ratio of the distances to find the new letter size.
- The ratio of the distances is 20 feet / 9.84 feet ≈ 2.03.
- Therefore, the letter size at 3 meters should be 17.46 mm / 2.03 ≈ 8.6 mm.

Step 5: Match with Options

- The closest option to 8.6 mm is **B- 8.9 mm**.

Final Answer

B- 8.9 mm

Deepseek R1 : Correct answer

17. What is the size of the smallest letter that a person with visual acuity LogMar0.3 will recognize from 3 meters?

A- 4.45 mm

B- 8.9 mm

C- 18.0 mm

D- 0.34 cm

Thought for 197 seconds

Okay, so I need to figure out the size of the smallest letter that a person with LogMAR 0.3 can recognize from 3 meters. The options are given in millimeters and centimeters. Let me start by recalling what LogMAR acuity means.

LogMAR stands for Logarithm of the Minimum Angle of Resolution. It's a measure of visual acuity where 0.0 corresponds to normal vision (20/20 Snellen). Each increase of 0.1 LogMAR represents a decrease in visual acuity by one line on an eye chart. So, a LogMAR of 0.3 would be worse than 20/20. Let me confirm: if LogMAR 0.0 is 20/20, then LogMAR 0.3 should be 20/(20*10^(0.3))? Wait, maybe I should convert LogMAR to Snellen first.

The formula to convert LogMAR to Snellen is Snellen acuity = 20 / (10^(LogMAR)). So if LogMAR is 0.3, then Snellen would be 20 / (10^0.3). Let me compute 10^0.3. Since 10^0.3 is the same as e^(0.3*ln10) ≈ e^(0.3*2.3026) ≈ e^0.6908 ≈ 1.995, which is approximately 2. So 10^0.3 ≈ 2. Therefore, Snellen acuity would be 20 / 2 = 10. So Snellen 20/10? Wait, that would be better than 20/20, which doesn't make sense because a LogMAR of 0.3 is worse than 0.0. Wait, maybe I have the formula backwards.

Wait, actually, the Snellen fraction is usually expressed as testing distance over the distance at which the letter subtends 5 minutes of arc. So, the LogMAR is the logarithm (base 10) of the minimum angle of resolution in minutes of arc. The formula is LogMAR = log10(minimum angle of resolution in minutes). For Snellen, 20/20 means that at 20 feet, the person can read letters that subtend 5 minutes of arc at 20 feet. If someone has worse vision, say 20/40, that means they can read letters at 20 feet that someone with normal vision can read at 40 feet, so the letters are twice as large, subtending 10 minutes of arc.

Wait, but LogMAR is calculated as log10(visual angle in minutes). So for 20/20, the visual angle is 5 minutes, so LogMAR = log10(5) ≈ 0.7? Wait, that can't be right. Wait, no. Wait, maybe I need to think differently.

Wait, actually, the standard for LogMAR is that each letter has a size that at a given distance subtends 5 minutes of arc, and each stroke width subtends 1 minute. So the LogMAR score is the log of the ratio of the observed threshold to the standard. So if someone can just read a letter that is twice as large (subtending 10 minutes of arc), then the LogMAR would be log10(10/5) = log10(2) ≈ 0.3. Wait, that would mean that a LogMAR of 0.3 corresponds to a visual acuity where the person needs letters twice as large as the standard. So that would correspond to Snellen 20/40, because 20/40 is twice the size of 20/20.

So if LogMAR 0.0 is 20/20, LogMAR 0.3 is 20/40. So, Snellen 20/40. Therefore, the question is, from 3 meters, what is the size of the smallest letter that a person with Snellen 20/40 can recognize?

So in Snellen terms, 20/40 means that the person can see at 20 feet what a normal person can see at 40 feet. So the letters need to be twice as large. If a normal person can see a certain size letter at 40 feet, this person needs those letters to be at 20 feet.

But here, the person is at 3 meters. Let me convert that to feet for Snellen. 3 meters is approximately 9.84 feet. Let's approximate it as 10 feet for simplicity. Wait, but maybe I need to work in meters. Let me check the standard.

In Snellen notation, 6/6 meters is normal vision (equivalent to 20/20 feet). So 6 meters is the standard. If someone has 6/12, that means they can see at 6 meters what a normal person can see at 12 meters. So the letters are twice as large.

So, for our case, converting LogMAR 0.3 to Snellen: as we thought, LogMAR 0.3 is equivalent to 20/40 (feet) or 6/12 (meters). So, if a person has 6/12 acuity, that means at 6 meters, they can read letters that a normal person can read at 12 meters. Therefore, the letters are twice as large as the standard.

But in this question, the person is at 3 meters. So, if the person's visual acuity is such that they need letters twice as large as normal, then at 3 meters, the letters they can read would be the same size as standard letters viewed at 6 meters. Wait, let me clarify.

Wait, if a normal person can read a certain letter size at 6 meters, a person with 6/12 acuity needs the letters to be twice as big (so that they can read them at 6 meters). Alternatively, if you test them at 3 meters, they could read letters that are half the size of the 6/6 letters. Wait, maybe not. Let me think again.

Visual acuity is about the angular size. The letter size is determined by the angle it subtends on the retina. The standard is that the letter's height subtends 5 minutes of arc, and the strokes subtend 1 minute of arc. If someone has worse acuity, they need a larger angle.

So, if the person's LogMAR is 0.3, that means their minimum resolvable angle is $10^{(LogMAR)} = 10^{0.3} \approx 2$ minutes of arc. Wait, no. Wait, LogMAR is defined as log10 of the minimum angle of resolution (MAR) in minutes. So if LogMAR is 0.3, then $MAR = 10^{0.3} \approx 2$ minutes of arc. Wait, but normal MAR is 1 minute of arc (for 20/20). So if someone has a MAR of 2 minutes, their acuity is worse. So the Snellen fraction would be 1/MAR. Wait, maybe.

Visual acuity is about the angular size. The letter size is determined by the angle it subtends on the retina. The standard is that the letter's height subtends 5 minutes of arc, and the strokes subtend 1 minute of arc. If someone has worse acuity, they need a larger angle.

So, if the person's LogMAR is 0.3, that means their minimum resolvable angle is $10^{(LogMAR)} = 10^{0.3} \approx 2$ minutes of arc. Wait, no. Wait, LogMAR is defined as log10 of the minimum angle of resolution (MAR) in minutes. So if LogMAR is 0.3, then $MAR = 10^{0.3} \approx 2$ minutes of arc. Wait, but normal MAR is 1 minute of arc (for 20/20). So if someone has a MAR of 2 minutes, their acuity is worse. So the Snellen fraction would be 1/MAR. Wait, maybe.

Wait, the Snellen fraction is test distance over the distance where the letter subtends 5 minutes of arc. So if a person has a MAR of 2 minutes, then the letter they can just resolve would subtend 2 minutes of arc at their testing distance. Therefore, the Snellen fraction would be testing distance / (testing distance * (MAR / 1)) = 1 / MAR. Wait, maybe not.

Wait, maybe it's better to use the formula: Snellen fraction = $20 / (10^{(LogMAR)})$. Wait, earlier I thought that but confused myself. Let me check with standard values. If LogMAR is 0.0, Snellen fraction is 20/20. If LogMAR is 0.3, then Snellen fraction is $20 / (10^{0.3}) \approx 20 / 2 \approx 10$, so 20/10? That would be better than normal, which contradicts. Wait, something's wrong here.

Wait, maybe the formula is inverted. Let me look up the conversion formula between LogMAR and Snellen.

Upon checking, the correct formula is:

Snellen fraction = $10^{(-LogMAR)}$

But that gives Snellen as a decimal. Wait, maybe not. Alternatively, the formula is:

LogMAR = log10(1 / Snellen)

If Snellen is expressed as a decimal fraction, e.g., 20/40 = 0.5. So LogMAR = log10(1 / 0.5) = log10(2) ≈ 0.3. Yes, that's correct. So Snellen 20/40 is 0.5, and LogMAR is log10(1/0.5) = 0.3. So the formula is LogMAR = log10(1 / (Snellen fraction)).

Therefore, if the LogMAR is 0.3, the Snellen fraction is 10^(-0.3) ≈ 0.501, which is approximately 20/40 (since 20/40 = 0.5). So Snellen equivalent is 20/40.

So, now, the question is, from 3 meters, what is the size of the smallest letter that a person with 20/40 vision can recognize?

In Snellen terms, 20/40 means that the person can see at 20 feet what a normal person sees at 40 feet. Therefore, the letters need to be twice as large. Translating this to metric, 6 meters is the standard. So 6/12 would be equivalent to 20/40. So a person with 6/12 vision needs letters twice as large as the standard 6/6 letters.

Now, the standard letter size for 6/6 (or 20/20) is such that the letter's height subtends 5 minutes of arc, and the critical detail (like the stroke width) subtends 1 minute of arc.

First, let's find the physical size of a standard letter (6/6) at 6 meters. The angular height is 5 minutes of arc. Let's convert that angle to radians to calculate the physical size.

One minute of arc is 1/60 of a degree. So 5 minutes is 5/60 = 1/12 degrees. Convert degrees to radians: 1 degree = π/180 radians. So 1/12 degrees is π/(180*12) ≈ 0.00014544 radians.

The formula for the size (height) of the letter is: angle (radians) = size / distance. Therefore, size = angle * distance.

At 6 meters, standard letter height is:

size = 0.00014544 radians * 6 meters ≈ 0.00087264 meters ≈ 0.87264 mm. Wait, that seems really small. Wait, no, 5 minutes of arc for the entire letter height. But maybe stroke width is 1 minute. Let me check again.

Wait, actually, in Snellen charts, the overall height of the letter is 5 times the stroke width. So if the stroke width subtends 1 minute of arc, the entire letter height subtends 5 minutes. So the total height is 5 times the stroke width. So perhaps the calculation should be done for the stroke width.

Wait, but the question is about the size of the letter. If the entire letter is 5 times the stroke width, then if the stroke width is 1 minute, the letter height is 5 minutes. So to calculate the letter height, it's 5 times the stroke width. So if we compute the height, it's 5 minutes of arc.

Therefore, let's compute the height of a standard 6/6 letter at 6 meters. 5 minutes of arc is 5/60 = 1/12 degree. Convert to radians: 1/12 * π/180 ≈ 0.00014544 radians. Then, size = 0.00014544 * 6 meters ≈ 0.00087264 meters, which is 0.87264 mm. Wait, that seems too small. Because in reality, Snellen letters are much larger. For example, the 20/20 letter is about 8.7 mm in height at 20 feet. Let me check.

Wait, 20 feet is approximately 6.096 meters. So, at 20 feet (6.096 meters), a 20/20 letter subtends 5 minutes of arc. Let's compute that. 5 minutes is 5/60 = 1/12 degree. Convert to radians: 1/12 * π/180 ≈ 0.00014544 radians. Then, size = 0.00014544 * 6.096 ≈ 0.000886 meters, which is approximately 0.886 cm or 8.86 mm. Ah, okay, so the standard 20/20 letter is about 8.7 mm at 20 feet (6 meters). So that's correct. So, at 6 meters, a 8.7 mm letter subtends 5 minutes of arc.

Therefore, a person with 20/40 (LogMAR 0.3) vision would need letters twice as large. So at 6 meters, they need letters that are 17.4 mm in height. But the question is asking from 3 meters. So if the person is at 3 meters, how big should the letters be?

Visual acuity is about angular size. So regardless of the distance, the angular size must meet their threshold. So if they have LogMAR 0.3, which corresponds to MAR 10^0.3 ≈ 2 minutes of arc. Wait, no, LogMAR is the logarithm of the MAR. So MAR = 10^(LogMAR). Wait, if LogMAR is 0.0, MAR is 1 minute (normal). If LogMAR is 0.3, MAR is 10^0.3 ≈ 2 minutes. Therefore, their MAR is 2 minutes. Since the letter height is 5 times the stroke width, which is 5 * MAR. Wait, but MAR is the minimum angle resolvable. So the stroke width of the letter must be equal to the MAR. Therefore, the letter height is 5 * MAR.

Therefore, the angular height of the letter is 5 * MAR. So for LogMAR 0.3, MAR is 10^0.3 ≈ 2 minutes. Therefore, the angular height is 5 * 2 = 10 minutes of arc. So the letter must subtend 10 minutes of arc at the eye.

So now, we need to find the physical height of a letter that subtends 10 minutes of arc at 3 meters.

First, convert 10 minutes to degrees: 10 / 60 = 1/6 degree. Convert to radians: 1/6 * π/180 ≈ 0.000290888 radians.

Then, physical height = angle (radians) * distance.

Distance is 3 meters. So:

height = 0.000290888 * 3 ≈ 0.000872664 meters ≈ 0.872664 cm ≈ 8.72664 mm.

So approximately 8.7 mm. Looking at the options: A is 4.45 mm, B is 8.9 mm, C is 18 mm, D is 0.34 cm (3.4 mm). So 8.7 mm is closest to option B (8.9 mm).

But wait, let me check my calculations again. Maybe I made an error.

Wait, LogMAR 0.3 corresponds to MAR = 10^0.3 ≈ 2 minutes. So the stroke width is 2 minutes. The letter height is 5 times the stroke width, so 10 minutes.

Calculating the height in mm at 3 meters:

Angle θ = 10 minutes = 10/60 = 1/6 degree. Convert to radians: 1/6 * π/180 ≈ 0.000290888 radians.

Height h = θ * distance. Distance is 3 meters = 3000 mm. Wait, wait, if distance is in meters and angle is in radians, height in meters. But if I use distance in millimeters, height will be in millimeters. Let's do that.

θ = 0.000290888 radians

Distance d = 3 meters = 3000 mm

h = θ * d = 0.000290888 * 3000 ≈ 0.872664 mm * 3000? Wait, no. Wait, θ is in radians, so h = θ * d. So:

h = 0.000290888 * 3000 ≈ 0.872664 mm? Wait, no. Wait, 0.000290888 * 3000 mm = 0.000290888 * 3000 = 0.872664 mm? That can't be right. Wait, that's 0.87 mm. But we know that at 6 meters, 5 minutes is 8.7 mm. So at 3 meters, 10 minutes should be equivalent to doubling the distance. Wait, maybe there's a miscalculation here.

Wait, maybe I confused meters and millimeters. Let's do it in meters:

θ = 10 minutes = 10/60 degrees = 1/6 degree. Convert to radians: 1/6 * π/180 ≈ 0.000290888 radians.

Distance = 3 meters.

h = θ * distance = 0.000290888 * 3 ≈ 0.000872664 meters ≈ 0.872664 millimeters. Wait, that's 0.87 mm. But that contradicts the earlier example where at 6 meters, 5 minutes is 8.7 mm.

Wait, hold on, if 5 minutes at 6 meters is 8.7 mm, then 10 minutes at 3 meters would be:

First, note that if you halve the distance, the same physical size would subtend twice the angle. So, if a letter is 8.7 mm at 6 meters subtends 5 minutes, then at 3 meters, it would subtend 10 minutes. Therefore, a letter of 8.7 mm at 3 meters would subtend 10 minutes. Therefore, the height needed is 8.7 mm, which is option B (8.9 mm).

Yes, this makes sense. Therefore, the answer should be B.

Alternatively, using the formula:

The standard letter height at 6 meters (20/20) is 8.7 mm. A person with 20/40 vision needs letters twice as large. So at 6 meters, they need 17.4 mm. But if they are at 3 meters, which is half the distance, the required letter size would be half of 17.4 mm, which is 8.7 mm. Hence, 8.7 mm, which is approximately 8.9 mm as in option B.

Yes, that's consistent. So the correct answer is B.

Final Answer

$$\boxed{B}$$

To determine the size of the smallest letter that a person with LogMAR 0.3 can recognize from 3 meters, we need to convert LogMAR to Snellen acuity and then calculate the letter size based on the visual angle.

1. **Convert LogMAR to Snellen Acuity**:
   - LogMAR 0.3 corresponds to a Snellen fraction of $10^{-0.3} \approx 0.5$, which is equivalent to 20/40 or 6/12. This means the person needs letters twice as large as those seen by a person with normal vision (20/20).

2. **Calculate the letter size**:
   - A person with 20/40 vision needs letters twice the size of the standard 20/20 letters.
   - The standard letter height for 20/20 vision at 6 meters is approximately 8.7 mm.
   - At 3 meters (half the distance), the required letter size would be half of twice the standard size. This is because halving the distance doubles the angular size, so the physical size needed is the same as the standard size at 6 meters.
3. **Convert the standard size to the required distance**:
   - The standard letter height at 6 meters (8.7 mm) subtends 5 minutes of arc.
   - At 3 meters, to subtend the same angle (10 minutes of arc for 20/40 vision), the letter size is calculated to be approximately 8.7 mm. This is closest to option B (8.9 mm).

Thus, the size of the smallest letter that a person with LogMAR 0.3 will recognize from 3 meters is $\boxed{B}$.

Gemini 2.0 Flash : Uncorrect answer

17. What is the
size of the smallest letter that a person with visual
acuity LogMar0.3 will recognize from 3 meters?
A- 4.45 mm
B- 8.9 mm
C- 18.0 mm
D- 0.34 cm

Here's how to calculate the size of the smallest letter a person with 0.3 LogMAR visual acuity can recognize from 3 meters:

Understanding LogMAR

- LogMAR (Logarithm of the Minimum Angle of Resolution) is a way to express visual acuity. A lower LogMAR value indicates better vision.
- A LogMAR of 0 means perfect vision (20/20 or 6/6). Each 0.1 increase in LogMAR represents a halving of visual acuity.

Calculation

1. **Convert LogMAR to MAR (Minimum Angle of Resolution):**
   - MAR = 10LogMAR
   - MAR = 100.3
   - MAR ≈ 2 minutes of arc
2. **Convert MAR to radians:**
   - MAR (in radians) = (MAR / 60) * (π / 180)
   - MAR (in radians) ≈ (2 / 60) * (π / 180)
   - MAR (in radians) ≈ 0.00058 radians
3. **Calculate letter size:**
   - Letter size = Viewing distance * tan(MAR in radians)
   - Letter size = 3 meters * tan(0.00058 radians)
   - Letter size ≈ 0.00174 meters
4. **Convert to millimeters:**
   - Letter size ≈ 0.00174 meters * 1000 mm/meter
   - Letter size ≈ 1.74 mm

Therefore, none of the provided multiple-choice options are correct. The closest answer would be approximately 1.74 mm.

This example question illustrates that while several chatbots ultimately arrived at the correct answer, their calculation process is often more elaborate and time-consuming than what is typically employed in clinical practice. In real-world settings, ophthalmologists frequently rely on straightforward tables and simplified formulas to compute the same parameter in a fraction of the time. This highlights an important dimension of clinical expertise: not only the ability to reach an accurate result, but also the capacity to do so rapidly and efficiently, a skill that is critical in high-pressure patient care environments.
